# The Fast-Growing *Brucella suis* Biovar 5 Depends on Phosphoenolpyruvate Carboxykinase and Pyruvate Phosphate Dikinase but Not on Fbp and GlpX Fructose-1,6-Bisphosphatases or Isocitrate Lyase for Full Virulence in Laboratory Models

**DOI:** 10.3389/fmicb.2018.00641

**Published:** 2018-04-05

**Authors:** Amaia Zúñiga-Ripa, Thibault Barbier, Leticia Lázaro-Antón, María J. de Miguel, Raquel Conde-Álvarez, Pilar M. Muñoz, Jean J. Letesson, Maite Iriarte, Ignacio Moriyón

**Affiliations:** ^1^Departamento de Microbiología y Parasitología e Instituto de Salud Tropical – Instituto de Investigación Sanitaria de Navarra, Universidad de Navarra, Pamplona, Spain; ^2^Research Unit in Biology of Microorganisms, Namur Research Institute for Life Sciences, University of Namur, Namur, Belgium; ^3^Unidad de Producción y Sanidad Animal, Instituto Agroalimentario de Aragón, Centro de Investigación y Tecnología Agroalimentaria de Aragón, Universidad de Zaragoza, Zaragoza, Spain

**Keywords:** *Brucella*, metabolism, gluconeogenesis, pyruvate phosphate dikinase, phosphoenolpyruvate carboxykinase, malic enzyme, isocitrate lyase

## Abstract

Bacteria of the genus *Brucella* infect a range of vertebrates causing a worldwide extended zoonosis. The best-characterized brucellae infect domestic livestock, behaving as stealthy facultative intracellular parasites. This stealthiness depends on envelope molecules with reduced pathogen-associated molecular patterns, as revealed by the low lethality and ability to persist in mice of these bacteria. Infected cells are often engorged with brucellae without signs of distress, suggesting that stealthiness could also reflect an adaptation of the parasite metabolism to use local nutrients without harming the cell. To investigate this, we compared key metabolic abilities of *Brucella abortus* 2308 Wisconsin (2308W), a cattle biovar 1 virulent strain, and *B. suis* 513, the reference strain of the ancestral biovar 5 found in wild rodents. *B. suis* 513 used a larger number of C substrates and showed faster growth rates *in vitro*, two features similar to those of *B. microti*, a species phylogenomically close to *B. suis* biovar 5 that infects voles. However, whereas *B. microti* shows enhanced lethality and reduced persistence in mice, *B. suis* 513 was similar to *B. abortus* 2308W in this regard. Mutant analyses showed that *B. suis* 513 and *B. abortus* 2308W were similar in that both depend on phosphoenolpyruvate synthesis for virulence but not on the classical gluconeogenic fructose-1,6-bisphosphatases Fbp-GlpX or on isocitrate lyase (AceA). However, *B. suis* 513 used pyruvate phosphate dikinase (PpdK) and phosphoenolpyruvate carboxykinase (PckA) for phosphoenolpyruvate synthesis *in vitro* while *B. abortus* 2308W used only PpdK. Moreover, whereas PpdK dysfunction causes attenuation of *B. abortus* 2308W in mice, in *B. suis*, 513 attenuation occurred only in the double PckA-PpdK mutant. Also contrary to what occurs in *B. abortus* 2308, a *B. suis* 513 malic enzyme (Mae) mutant was not attenuated, and this independence of Mae and the role of PpdK was confirmed by the lack of attenuation of a double Mae-PckA mutant. Altogether, these results decouple fast growth rates from enhanced mouse lethality in the brucellae and suggest that an Fbp-GlpX-independent gluconeogenic mechanism is ancestral in this group and show differences in central C metabolic steps that may reflect a progressive adaptation to intracellular growth.

## Introduction

In order to survive and efficiently replicate, pathogens need to adjust their metabolism to the nutrients available in their hosts. This is the case of the brucellae, a group of Gram-negative bacteria that infect a wide range of vertebrates (Whatmore, 2009; Zheludkov and Tsirelson, 2010; Soler-Lloréns et al., 2016; Al Dahouk et al., 2017) and include facultative intracellular pathogens causing brucellosis, a worldwide distributed zoonosis. Taxonomically, these bacteria are grouped currently in a single genus with up to 12 closely related species^1^, some of which were divided long ago into biovars according to phenotypic criteria (Alton et al., 1988). Recent studies show that most brucellae form a core group, which includes the “classical” species as well as more recent isolates from a variety of mammals, separated from several early diverging brucellae, which in turn are close to environmental bacteria and opportunistic pathogens of the α-2 Proteobacteria (Soler-Lloréns et al., 2016; Al Dahouk et al., 2017). Thus far, the core brucellae that infect domestic ruminants (*Brucella abortus* and *B. melitensis*) and swine (*B. suis* biovars 1, 2, and 3) have deserved greater attention undoubtedly because of their early identification and great impact on public health and animal production. Even though these three species are often described as fastidious because of their slow growth and complex requirements for primary isolation (peptone-yeast extract media, often supplemented with serum), under laboratory conditions the strains investigated are auxotrophic for a few vitamins and, but for some strains that seem to require some amino acids (Plommet, 1991; see also section “Discussion”), they grow on mineral salts with glutamate-lactate-glycerol or glucose (Gerhardt and Wilson, 1948; Plommet, 1991; Barbier et al., 2018). However, there is only limited information on the substrates and pathways in their replicative niche, a vacuole connected to ER cisternae and the outer nuclear membrane (Pizarro-Cerdá et al., 1998; Starr et al., 2008; Ronneau et al., 2014; Zúñiga-Ripa et al., 2014; Barbier et al., 2018; Sedzicki et al., 2018).

**FIGURE 1:**
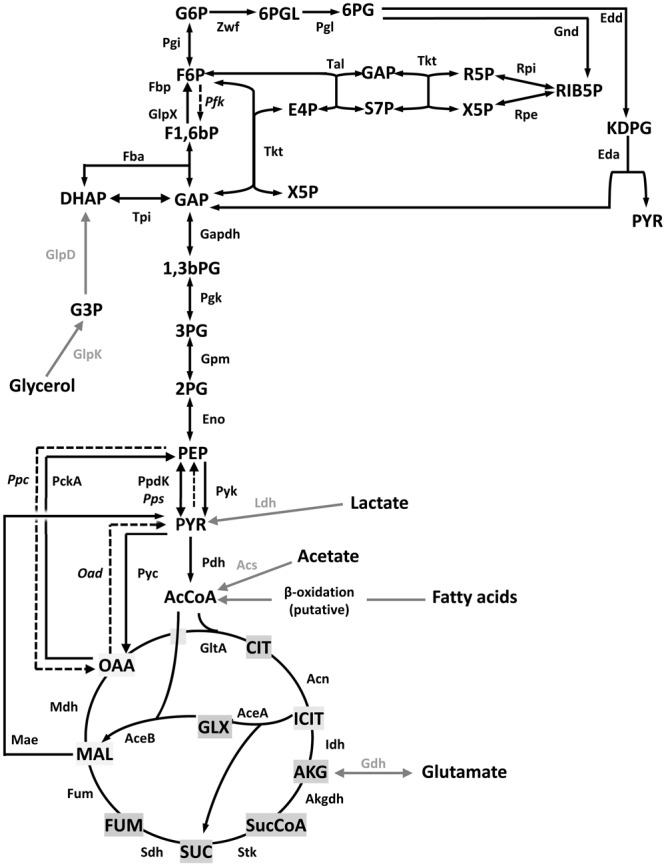
Central C metabolic network of *Brucella* (adapted from Zúñiga-Ripa et al., 2014). The metabolic network includes complete pentoses phosphate, Entner–Doudoroff and gluconeogenesis pathways, as well as a complete tricarboxylic acid cycle including a glyoxylate shunt. The Embden–Meyerohof–Parnas pathway is interrupted due to the lack of phosphofructokinase (Pfk). Black dashed arrows and italics indicate steps for which no putative genes can be identified in *Brucella* Gray arrows and gray font indicate peripheral pathways. Metabolites: 1,3,bPG, 1,3-bisphosphoglycerate; KDPG, 2-keto-3- deoxy-phosphogluconate; 2PG, 2-phosphoglycerate; 3PG, 3-phosphoglycerate; 6PGL, 6-P-gluconolactone; 6PG, 6-phosphogluconate; AcCoA, acetyl-coenzyme A; AKG, alpha-ketoglutarate; CIT, citrate; ICIT, isocitrate; DHAP, dihydroxyacetone-phosphate; E4P, erythrose-4-phosphate; F1,6bP, fructose-1,6-bisphosphate; F6P, fructose-6-phosphate; FUM, fumarate; G6P, glucose-6-P; GAP, glyceraldehyde-3-phosphate; G3P, glycerol-3-phosphate; GLX, glyoxylate; MAL, malate; OAA, oxaloacetate; PEP, phosphoenolpyruvate; PYR, pyruvate; R5P, ribose-5-P; RIB5P, ribulose-5-P; S7P, sedoheptulose-7-P; SUC, succinate; SucCoA, succinyl-coenzyme A; X5P, xylulose-5-P. Enzymes: Edd, 6-phospho-D-gluconate dehydratase; Gnd, 6-phosphogluconate dehydrogenase; Pgl, 6-phosphogluconolactonase; Acs, acetyl-coenzyme A synthetase; Acn, aconitate hydratase; Akgdh, alpha-ketoglutarate dehydrogenase; GltA, citrate synthase; eno, enolase; Fbp, GlpX, fructose-1,6-bisphosphatase; Fba, fructose bisphosphate aldolase; Fum, fumarase; Zwf, glucose-6-phosphate dehydrogenase; Pgi, glucose-6-phosphate isomerase; Gdh, glutamate dehydrogenase; Gapdh, glyceraldehyde-3-phosphate dehydrogenase; GlpD, glycerol-3-phosphate dehydrogenase; GlpK, glycerol kinase; Idh, isocitrate dehydrogenase; AceA, isocitrate lyase; Eda, 2-dehydro-3-deoxy-phosphogluconate aldolase; Ldh, lactate dehydrogenase; Mdh, malate dehydrogenase; AceB, malate synthase; Mae, malic enzyme; PckA, phosphoenolpyruvate carboxykinase; Ppc, phosphoenolpyruvate carboxylase; Pps, phosphoenolpyruvate synthase; Pfk, phosphofructokinase; Pgk, phosphoglycerate kinase; Gpm, phosphoglycerate mutase; Oad, pyruvate carboxykinase; Pyc, pyruvate carboxylase; Pdh, pyruvate dehydrogenase; Pyk, pyruvate kinase; PpdK, pyruvate phosphate dikinase; Rpi, ribose-5-phosphate isomerase; Rpe, ribulose-5-phosphate-3-epimerase; Sdh, succinate dehydrogenase; Stk, succinyl-coenzyme A synthethase; Tal, transaldolase; Tkt, transketolase; Tpi, triose phosphate isomerase.

The central C metabolism pathways of *Brucella* have been reviewed recently (Barbier et al., 2018). Radiorespirometric and biochemical analyses show that *B. suis* 1330 (reference strain of biovar 1), *B. melitensis* 16M (reference strain of biovar 1) and *B. abortus* 2308 [biovar 1, National Animal Disease Laboratory (Ames, IA, United States)] and S19 (attenuated vaccine strain) can split hexoses into trioses (Robertson and McCullough, 1968). However, there is no phosphofructokinase (Pfk; **Figure 1**) and glycolysis [i.e., the Embden–Meyerhof–Parnas (EMP)] pathway is thus interrupted. Similarly, although all genes of the Entner–Doudoroff (ED) pathway are present, the dehydratase (Edd) activity could not be detected in the strain tested (S19). Accordingly, the pentose shunt would be the only route that can provide phosphorylated trioses for subsequent oxidation in the tricarboxylic acid (TCA) cycle (Barbier et al., 2018; **Figure 1**). Surprisingly, a *B. abortus* 2308 Wisconsin (2308W; see Supplementary Table S1 and Suárez-Esquivel et al., 2016) double *fbp* and *glpX* mutant (the canonical gluconeogenic fructose-1,6-bisphosphatase genes; **Figure 1**) grows in gluconeogenic media, albeit at a markedly reduced rate (Zúñiga-Ripa et al., 2014). Moreover, attenuation in BALB/c mice was observed for pyruvate phosphate dikinase (PpdK) and malic enzyme (Mae) mutants but not for mutants in Fbp, GlpX, phosphoenolpyruvate carboxykinase (PckA) or isocitrate lyase (AceA; glyoxylate shunt) (**Figure 1**). These observations suggest that *B. abortus* 2308W is endowed with unconventional gluconeogenic enzymes and that, during infection, has access to a limited supply of 6 and 5 C substrates that is compensated through anaplerotic routes by TCA intermediates without a critical role of the glyoxylate shunt.

Phylogenomic analyses show that the core brucellae are less uniform than previously assumed on the basis of DNA:DNA hybridization (Verger et al., 1985). While all *B. abortus* and *B. melitensis* biovars group into two clades, the five recognized biovars of *B. suis* show phylogenomic diversity inconsistent with their grouping into a single species (Moreno et al., 2002; Scholz et al., 2008; Whatmore, 2009; Al Dahouk et al., 2012) and a very wide host range. Biovars 1 and 3 of this nominal species infect swine in the countries of America, Asia, and Europe; biovar 2, swine and hares in Europe; biovar 4, Artic and Northern Eurasia reindeers; and biovar 5 were isolated from species of wild rodents in Transcaucasia some 30 years ago (Zheludkov and Tsirelson, 2010). Since *B. suis* biovar 5 (reference strain 513) is closer to the ancestral brucellae (Whatmore, 2009; Al Dahouk et al., 2017), in this work, we examined whether it shares with *B. abortus* some relevant nutritional characteristics and steps of the central C metabolism, an information that may help to identify pathways that are conserved and may thus be important in the intracellular life of core brucellae. Here, we report similarities and differences and discuss their potential significance in the lifestyle of these bacteria. The differences and similarities between *B. suis* 513 and *B. microti* (a species isolated more recently from *Microtus arvalis*), noticed in the course of this study are also discussed.

## Materials and Methods

### Bacterial Strains and Growth Conditions

The bacterial strains and plasmids used in this study are listed in Supplementary Table S1. All *Brucella* were handled under BSL-3 containment. The strains resulting from the genetic manipulations described below were characterized according to standard *Brucella* typing procedures (Alton et al., 1988): colonial morphology after 3 days of incubation at 37°C, crystal violet exclusion, urease, acriflavine agglutination, sensitivity to Tb, Wb, Iz, and R/C phages, agglutination with anti-A and anti-M monospecific sera, CO_2_ and serum dependence, and susceptibility to thionine blue, fuchsine, and safranin. Bacteria were routinely grown in standard Peptone-Glucose [Biomerieux; bio-Trypcase (17 g/L), bio-Soyase (3 g/L), Glucose (2.5 g/L), NaCl (5 g/L), K_2_HPO_4_ (2.5 g/L)] or this media supplemented with agar. The Peptone-Yeast Extract medium used was composed of bacto tryptone (16 g/L), yeast extract (10 g/L), and NaCl (5 g/L) (all from BD Difco). The following antibiotics were used at the indicated concentrations: kanamycin (Km; 50 μg/mL), polymyxin (Pmx; 1.5 μg/mL), and/or chloramphenicol (Cm; 20 μg/mL) (all from Sigma). When needed, media was supplemented with 5% sucrose. All strains were stored in skimmed milk at -80°C (Scharlau).

To study the phenotype of the metabolic mutants constructed, the defined medium of Gerhardt (glutamate-lactate-glycerol) was used (Gerhardt and Wilson, 1948). The components for 1 L medium are: glycerol (30 g), lactic acid (5 g), glutamic acid (1.5 g), thiamine (0.2 mg), nicotinic acid (0.2 mg), pantothenic acid (0.04 mg), biotin (0.0001 mg), K_2_HPO_4_ (10 g), Na_2_S_2_O_3_⋅5H_2_O (0.1 g), MgSO_4_ (10 mg), MnSO_4_ (0.1 mg), FeSO_4_ (0.1 mg), and NaCl (7.5 g). The pH was adjusted to 6.8–7. In addition, a modification of Plommet’s medium was also used (Plommet, 1991; Barbier et al., 2014) and 1 L of this medium is composed of thiamine (0.2 g), nicotinic acid (0.2 g), pantothenic acid (0.07 g), biotin (0.1 mg), K_2_HPO_4_ (2.3 g), KH_2_PO_4_ (3 g), Na_2_S_2_O_3_ (0.1 g), MgSO_4_ (0.01 g), MnSO_4_ (0.1 mg), FeSO_4_ (0.1 mg), NaCl (5 g), (NH_4_)_2_SO_4_ (0.5 g), and 1 g/L of substrate. When glutamic acid was used as nitrogen and C source (NH_4_)_2_SO_4_ was not added.

### DNA Manipulations

Genomic sequences of the different *Brucella* species were obtained from the database National Center for Biotechnology Information (NCBI) and Kyoto Encyclopedia of Genes and Genomes (KEGG). Searches for DNA and protein homologies were carried out using NCBI BLAST (Altschul et al., 1990). Sequence alignments were performed with Clustal Omega (Goujon et al., 2010; Sievers et al., 2011). Primers were synthesized by Sigma (Haverhill, United Kingdom). DNA sequencing analysis was performed by the Servicio de Secuenciación de CIMA (Centro de Investigación Médica Aplicada, Universidad de Navarra, Pamplona, Spain). Restriction modification enzymes were used under the conditions recommended by the manufacturer. Plasmid and chromosomal DNA were extracted with QIAprep Spin Miniprep (Qiagen) and Ultraclean Microbial DNA Isolation kit (Mo Bio Laboratories), respectively. When needed, DNA was purified from agarose gels using QIAquick Gel Extraction Kit (Qiagen).

### Mutagenesis

Construction of the in-frame deletion mutants *Bs5Δfbp, Bs5ΔglpX, Bs5ΔfbpΔglpX, Bs5ΔpckA, Bs5ΔppdK, Bs5ΔpckA ΔppdK, Bs5Δmae, Bs5ΔmaeΔpckA*, and *Bs5ΔaceA* was done using previously described plasmids and strategy (Zúñiga-Ripa et al., 2014).

*Bs5ΔBMI_I149* (and *Bs5ΔmaeBs5ΔBMI_I149*) was obtained using the plasmid pAZI-25 constructed in this work. First, two PCR fragments were generated: oligonucleotides BMI_I149-F1 (5′-GGTTCCGGCTCTTTCTCTTC-3′) and BMI_I149-R2 (5′-AAAGTCGAGCGCTTCCTTCT-3′) amplified a 266 bp fragment including codons 1–31 of BMI_I149, as well as 161 bp upstream of the BMI_I149 start codon; oligonucleotides BMI_I149-F3 (5′-AGAAGGAAGCGCTCGACTTTAACCCGAAACTGATGGAACA-3′) and BMI_I149-R4 (5′-TGGACTTGCGATGACAGAAC-3′) were used to amplify a 356 bp fragment including the last 240 bp of BMI_I149. A third PCR joined the two fragments together using oligonucleotides BMI_I149-F1 and BMI_I149-R4 for amplification and the complementary regions between BMI_I149-R2 and BMI_I149-F3 for overlapping. The resulting fragment, containing the BMI_I149 deletion allele, was cloned into pCR2.1 (Invitrogen). After sequence verification, the insert was excised as a *Bam*HI–*Xba*I fragment and cloned in a pJQKm suicide vector (Scupham and Triplett, 1997). The acquisition of this vector by *Brucella* after mating with conjugative *Escherichia coli* S17 λpir was selected by Km and Pmx resistance. The loss of the plasmid concomitant with either a deletion or a return to wild type phenotype was then selected on sucrose. The resulting colonies were screened by PCR with primers BMI_I149-F1 and BMI_I149-R4 which amplified a fragment of 622 bp in the mutant and a fragment of 2599 bp in the revertant strain. To check the mutation, an internal primer (BMI_I149-R5) which hybridized in the non-deleted region was used.

For complementation, the plasmid pAZI-19 previously described (Zúñiga-Ripa et al., 2014) was used.

### Growth Curves

Growth curves were obtained using a Bioscreen C (Lab Systems) apparatus. To avoid carry over of media by the inoculum and lengthy lag phases, inocula were obtained from bacteria grown in test media as follows. First, the strains were inoculated into 10 mL of peptone-glucose in a 50 mL flask and incubated at 37°C with orbital shaking for 18 h. Then, these bacteria were harvested by centrifugation, resuspended in 10 mL of the test medium (peptone-glucose, peptone-yeast extract, glutamate-lactate-glycerol, glutamate, or lactate) at an OD_600 nm_ of 0.1, and incubated at 37°C with orbital agitation for 18 h. These exponentially growing bacteria were harvested by centrifugation, resuspended at an OD_600 nm_ of 0.1 in the test medium, dispensed as technical replicates in Bioscreen multiwell plates (200 μL/well; starting OD of 0.05 in the Bioscreen apparatus) and cultivated for 5 days with continuous shaking at 37°C. Absorbance values at 420–580 nm were automatically recorded at 30-min intervals. All experiments were repeated at least three times. Controls with medium and no bacteria were included in all experiments.

### Cell Culture and Infection

RAW 264.7 murine macrophages (ATCC TIB-71) were routinely cultured in Dulbecco’s Modified Eagle Medium (DMEM; Gibco) with 10% (vol/vol) heat-inactivated fetal bovine serum (Gibco), 1% (vol/vol) L-glutamine (200 nM; Sigma-Aldrich), and 1% (vol/vol) non-essential amino acids (Gibco). Cells were maintained at 37°C with a 5% CO_2_ atmosphere.

Infections were performed as previously described (Zúñiga-Ripa et al., 2014). Briefly, cells were seeded in 24-well plates at an appropriate density (1 × 10^5^ cells/well) and infected 24 h later with a multiplicity of infection (MOI) of 50:1. Cells were centrifuged at 400 × *g*, for 10 min at 4°C before being incubated for 15 min at 37°C with 5% CO_2_, washed with fresh medium and incubated for 90 min with medium containing 100 μg/mL of gentamycin. The medium was then replaced by a fresh one containing 25 μg/mL of this antibiotic. At each time point, cells were washed three times with 100 mM PBS (pH 7) before processing, lysed with 0.1% (v/v) Triton X-100 in PBS, and plated on peptone-glucose-agar to determine the number of intracellular bacteria. All experiments were performed in triplicate and results are expressed as means and standard errors (*n* = 3) of individual log_10_ CFU/well. The attenuated *B. abortus virB* mutant was used as a control (Sieira et al., 2000).

### Virulence Assays in Mice

Seven-week-old female BALB/c mice (Harlan Laboratories, Bicester, United Kingdom) were accommodated in the facilities of Centro de Investigación y Tecnología Agroalimentaria de Aragón (CITA; Registration code ES502970012025) for 2 weeks before and during the experiments, with water and food *ad libitum* under P3 biosafety containment conditions. The animal handling and other procedures were in accordance with the current European (directive 86/609/EEC) and Spanish (RD 53/2013) legislations, supervised by the Animal Welfare Committee of the CITA (2014-20).

For each strain, inoculum was prepared from cultures on peptone-glucose-agar at 37°C. Bacteria were harvested in 10 mM PBS (pH 7), suspended in this diluent to the appropriate concentration and approximately 5 × 10^4^ CFU in 0.1 mL administered to each mouse intraperitoneally (exact doses were retrospectively assessed). For each strain, mice (*n* = 5 per group) were inoculated and the CFU in spleens was determined at different weeks post-inoculation. Mice were anesthetized by intraperitoneal injection and sacrificed by cervical dislocation; spleens were isolated, weighted, homogenized in 9 vol of PBS and CFU counted on peptone-glucose-agar. The identity of the spleen isolates was confirmed by PCR at several points during the infection process. The individual data were normalized by logarithmic transformation, and the mean log_10_ CFU/spleen values and the standard deviation (*n* = 5) were calculated. Statistical significance was evaluated using one-way ANOVA followed by Dunnett’s test (^∗^*p* < 0.05, ^∗∗^*p* < 0.01, ^∗∗∗^*p* < 0.001, ^∗∗∗∗^*p* < 0.0001).

## Results

### *Brucella suis* 513 (Reference Strain of Biovar 5) Uses a Broad Range of Substrates as the Only C Source but It Is Not Prototrophic for Vitamins

**FIGURE 2:**
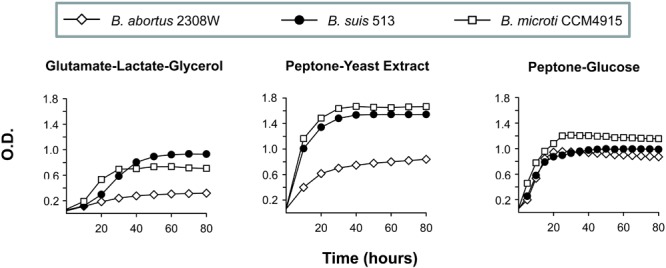
Growth curves in glutamate-lactate-glycerol, peptone-yeast extract, and peptone-glucose of *B. abortus* 2308W, *B. suis* 513, and *B. microti* CCM4915. Each point represents the mean ± standard error (error bars are within the size of the symbols) of optical density (OD) values of triplicate samples. The experiment was repeated three times with similar results.

**FIGURE 3:**
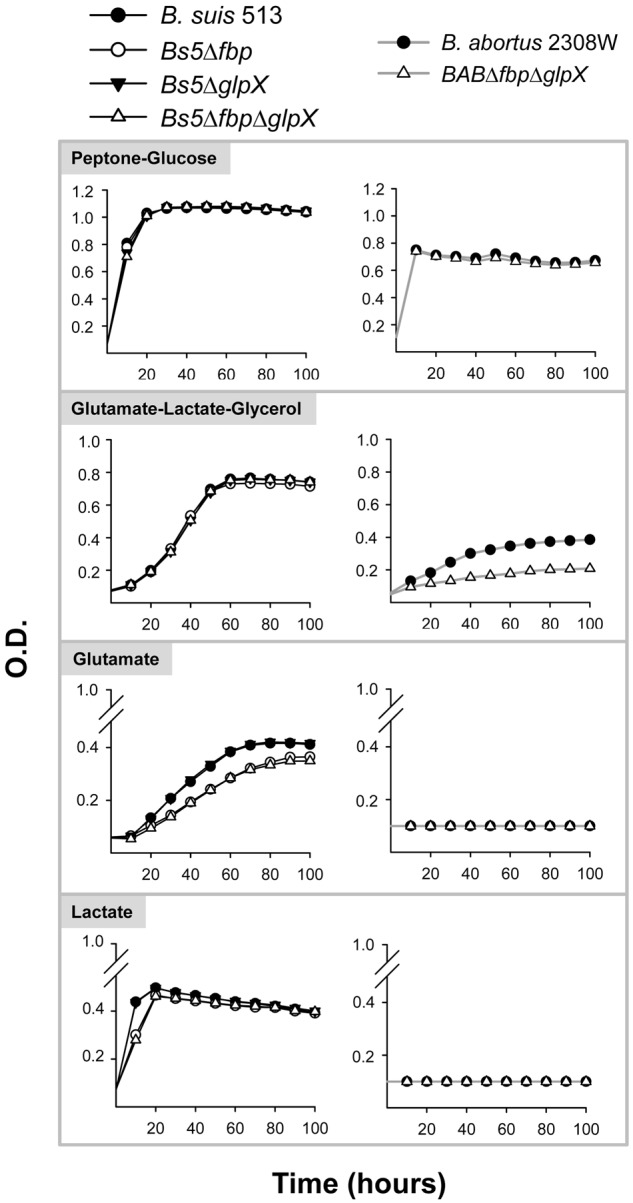
Growth curves in peptone-glucose, glutamate-lactate-glycerol, glutamate, and lactate of *B. suis* 513 and mutants *Bs5Δfbp, Bs5ΔglpX*, and *Bs5ΔfbpΔglpX*, and *B. abortus* 2308W and mutant *BABΔfbpΔglpX.* Each point represents the mean ± standard error (error bars are within the size of the symbols) of an experiment performed in technical triplicates. The experiment was repeated three times with similar results.

**FIGURE 4:**
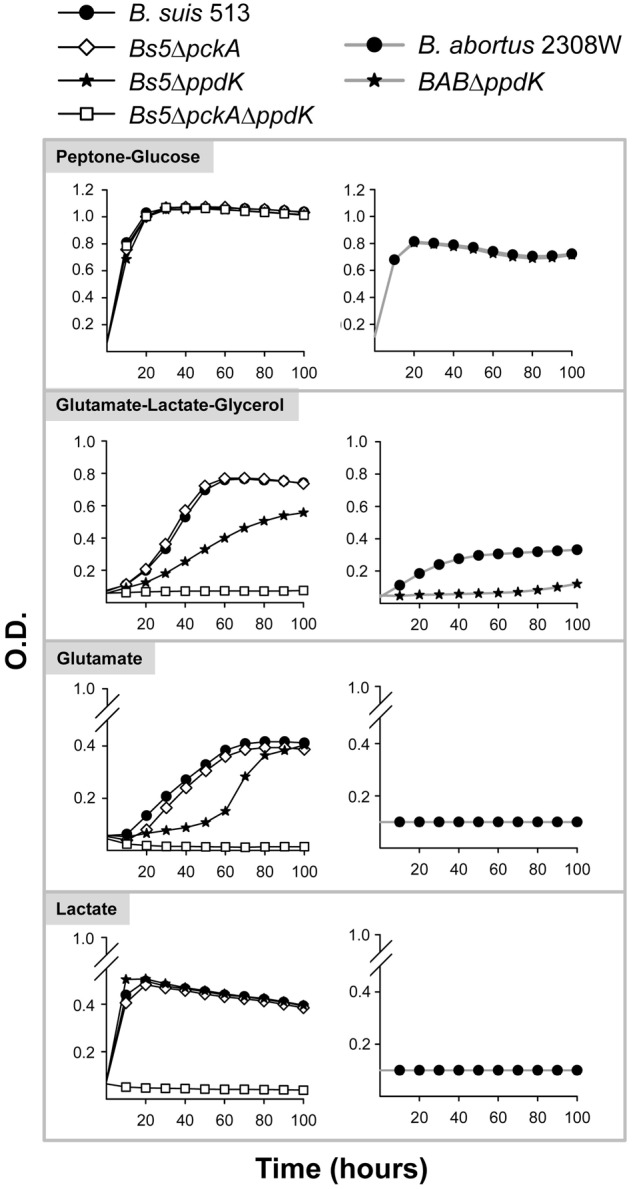
Growth curves in peptone-glucose, glutamate-lactate-glycerol, glutamate, and lactate of *B. suis* 513 and mutants *Bs5ΔpckA, Bs5ΔppdK*, and *Bs5ΔpckAΔppdK*, and *B. abortus* 2308W and mutant *BABΔppdK*. Each point represents the mean ± standard error (error bars are within the size of the symbols) of an experiment performed in technical triplicates. The experiment was repeated three times with similar results.

**FIGURE 5:**
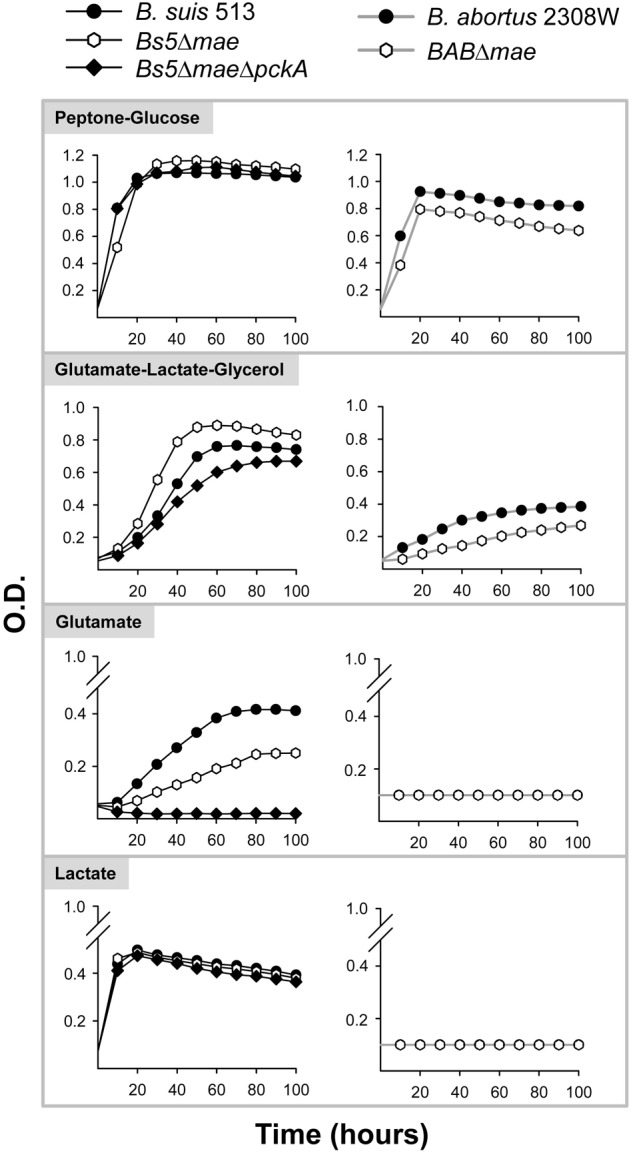
Growth curves in peptone-glucose, glutamate-lactate-glycerol, glutamate, and lactate of *B. suis* 513 and mutants *Bs5Δmae* and *Bs5ΔmaeΔpckA*, and *B. abortus* 2308W and mutant *BABΔmae.* Each point represents the mean ± standard error (error bars are within the size of the symbols) of an experiment performed in technical triplicates. The experiment was repeated three times with similar results.

Since the growth characteristics and requirements of *B. suis* 513 are practically unknown, we first examined the ability of this reference strain to grow on common C6, C5, C4, and C3 substrates as the only C source using the vitamins–mineral salts (with ammonium sulfate as N source when necessary) basal medium of Plommet (Plommet, 1991; **Table 1** and **Figures 2–5**). We found that the range of substrates used by *B. suis* 513 as the only C source was broader than that of *B. abortus* 2308W (Suárez-Esquivel et al., 2016). Remarkably, whereas *B. suis* 513 was able to grow efficiently on lactate or glutamate, *B. abortus* 2308W was not (**Table 1** and **Figures 3–5**) and required at least one additional C source (i.e., glutamate-lactate or glutamate-glycerol; not shown and Zúñiga-Ripa et al., 2014). These differences suggested that *B. suis* 513 is more prototrophic than *B. abortus* 2308W (see section “Discussion”), and thus we examined the vitamin requirements of *B. suis* 513. We found that *B. suis* 513 required nicotinic acid and thiamine (Supplementary Figure S1), being in this regard not different from *B. abortus, B. melitensis*, or the *B. suis* biovars previously studied, all of which require at least these two vitamins (Koser et al., 1941; Koser and Wright, 1942; McCullough and Dick, 1942a,b; McCullough et al., 1946).

**Table 1:** Growth of *B. abortus* 2308W and *B. suis* 513 on single C6, C4, and C3 compounds as the only C source^1^.

	Yield (OD_600 nm_)^2^	
C source	*B. abortus* 2308W	*B. suis* 513
**C6**		
Glucose	0.00	0.75
Fructose	0.00	0.25
Gluconate	0.00	0.00
Mannose	0.00	0.85
Fucose	0.00	0.60
Inositol	0.00	0.00
**C5**		
Ribose	0.11	0.90
Xilose	0.19	0.85
Glutamate	0.00	0.60
**C4**		
Succinate	0.00	0.00
Erythritol	0.70	0.90
**C3**		
Glycerol	0.00	0.00
Lactate	0.00	0.60
Pyruvate	0.00	0.70

*^*1*^C sources (1 g/L) were added to the vitamin-salt broth of Plommet’s medium (see section “Materials and Methods”). ^*2*^Yields correspond to the stationary phase (obtained at different times depending on the C source).*

### *Brucella suis* 513 Shows Faster Growth Rates Than *B. abortus* 2308W in Gluconeogenic Media That Do Not Depend on Fbp and GlpX

Since the above observations show that we could probe some aspects of the central C pathways of *B. suis* 513 using simple defined media, we compared first the growth of *B. suis* 513 with *B. abortus* 2308W (slow growing) and *B. microti* CCM4915 (usually described as fast growing) under gluconeogenic conditions (Gerhardt’s medium, containing glutamate, lactate, glycerol, mineral salts, and vitamins; henceforth glutamate-lactate-glycerol). As can be seen in **Figure 2**, the growth curves suggested higher growth rates for *B. suis* 513 and *B. microti* CCM4915 depending upon the medium. In peptone-yeast extract, a rich but still gluconeogenic medium where growth factors are abundant, *B. suis* 513 and *B. microti* CCM4915 displayed shorter generation times and much higher yields than *B. abortus* 2308W. These differences almost disappeared when peptone was combined with glucose (**Figure 2**). Taken together, these results suggest that *B. suis* 513 and *B. microti* CCM4915, on one hand, and *B. abortus* 2308W, on the other, differ in gluconeogenic abilities, a hypothesis that was examined in the experiments described below.

The brucellae carry homologs of the two canonic fructose-1,6-bisphosphatases (Fbp and GlpX) on which the gluconeogenic pathway depends. Consistent with our previous report (Zúñiga-Ripa et al., 2014), the simultaneous dysfunction of Fbp and GlpX did not affect the growth of *B. abortus* 2308W in peptone-glucose and impaired but did not abrogate its growth in glutamate-lactate-glycerol (**Figure 3**). In contrast, growth of single (*Bs5Δfbp* or *Bs5ΔglpX*) or double (*Bs5ΔfbpΔglpX*) bisphosphatase mutants of *B. suis* 513 was not compromised to any extent in glutamate-lactate-glycerol (**Figure 3**). Since *B. suis* 513 grew on lactate or glutamate as the sole C source, we also tested the mutants on these gluconeogenic substrates. We found that the ability to grow was not affected on lactate, and only slightly for *Bs5Δfbp* and *Bs5ΔfbpΔglpX* but not for *Bs5ΔglpX* on glutamate (**Figure 3**).

### *Brucella suis* 513 and *B. abortus* 2308W Differ in Key Steps Connecting Phosphoenolpyruvate and TCA

We then investigated whether growth of *B. suis* 513 in complex and gluconeogenic media depends on the steps catalyzed by PpdK (phosphoenolpyruvate ↔ pyruvate) and PckA (oxaloacetate → phosphoenolpyruvate) (**Figure 1**). As observed for *B. abortus* 2308W (**Figure 4** and Zúñiga-Ripa et al., 2014), deletion of *ppdK* (*Bs5ΔppdK*) impaired the growth of *B. suis* 513 on glutamate-lactate-glycerol but not on peptone-glucose (**Figure 4**). On the other hand, deletion of *pckA* had no effect in *B. suis* 513 (mutant *Bs5ΔpckA*; **Figure 4**) or in *B. abortus* 2308W (Zúñiga-Ripa et al., 2014). Although these observations could be interpreted to mean that, like in *B. abortus* 2308W (Zúñiga-Ripa et al., 2014), PckA is not functional and gluconeogenesis depends only on PpdK in *B. suis* 513, we found this hypothesis not to be true because growth on glutamate-lactate-glycerol was abrogated in a double *Bs5ΔpckAΔppdK* mutant (**Figure 4**), indicating that PckA is active in this strain. These results are in keeping with the fact that while *B. abortus* 2308W *pckA* carries a frameshift that generates a premature stop codon (TGA) at position 1474–1476, the orthologous codon in *B. suis* 513 is TGG (tryptophan) (a BLAST search did not reveal any other copy of *pckA* in the *B. abortus* 2308W genome).

We further explored the role of PpdK and PckA by growing *Bs5ΔppdK, Bs5ΔpckA*, and *Bs5ΔpckAΔppdK* on lactate or glutamate. But for the expected reduction in growth yields, the results obtained in glutamate paralleled those in glutamate-lactate-glycerol (**Figure 4**), confirming that the steps catalyzed by PckA and PpdK set a clear difference in the metabolism of *B. abortus* 2308W and *B. suis* 513 in gluconeogenic substrates. Interestingly, growth of *Bs5ΔppdK* in lactate was unaffected. As the brucellae lack gluconeogenic phosphoenolpyruvate synthase (Pps; **Figure 1**; Barbier et al., 2018), this result means that TCA cycle intermediates derived from the pyruvate obtained from lactate (**Figure 1**) can sustain gluconeogenesis. Since this could implicate a malic enzyme (Mae in **Figure 1**), we investigated the *mae* homologs of *B. suis* 513.

First, we identified a clear homolog of *B. abortus* 2308W *mae* in *B. suis* 513, which is also a homolog of *B. microti* (strain CCM4915) BMI_I1020. Although in *B. microti* there is a second ORF annotated as *mae* (BMI_I149), its *B. suis* 513 counterpart lacks a thymine at position 1153 that originates a frameshift that could compromise the functionality of the protein. Thus, we started studying the role of the first *mae* identified. Consistent with the presence of an active enzyme furnishing pyruvate from oxaloacetate (**Figure 1**), this *Bs5Δmae* mutant displayed a reduction in growth on glutamate but not on lactate, a phenotype similar to that of its *B. abortus* 2308W counterpart but for the expected differences in growth rates/yields (**Figure 5**). Moreover, growth on glutamate was abrogated in a double *Bs5ΔmaeΔpckA* mutant (**Figure 5**), the expected result if both Mae and PckA are active and the former acts in tandem with PpdK in gluconeogenesis (**Figure 1**). On the other hand, these mutants grew normally on lactate (**Figure 5**), as expected if lactate provides pyruvate for both gluconeogenesis and TCA reactions (**Figure 1**). Indirectly, the lack of growth of the double mutant on glutamate was coherent with the possibility that the frameshift in the BMI_I149 ortholog results in a non-functional protein, and we confirmed this using a double mutant in the *B. suis* 513 orthologs of BMI_I1020 (*mae*) and BMI_I149. This double mutant displayed the same growth characteristics as the single *Bs5Δmae* (Supplementary Figure S2), strongly suggesting that the protein encoded by the BMI_I149 homolog lacks Mae activity in *B. suis* 513.

The results presented above show that *B. suis* 513 converts malate to pyruvate via Mae, and oxaloacetate to phosphoenolpyruvate via PckA. Because these TCA cycle intermediates could be replenished by condensation of acetyl-CoA with glyoxylate into malate, we constructed an isocitrate lyase (AceA; **Figure 1**) mutant for *in vivo* studies (see below) on the role of the glyoxylate bypass. As expected, growth of this *B. suis* 513 mutant was identical to that of the parental strain in peptone-yeast extract, and this mutation had no effect on the growth in glutamate-lactate-glycerol, glutamate-glycerol, or lactate-glycerol (results not shown). Similarly, growth was not affected in media containing peptones (not shown), even though acetogenic amino acids (leucine, isoleucine, lysine, phenylalanine, tryptophan, and tyrosine) represent approximately 20% of the peptone amino acid content.

### *Brucella suis* 513 Requires *pckA* or *ppdK* but Not *mae* or *aceA* for Virulence in Mice

**FIGURE 6:**
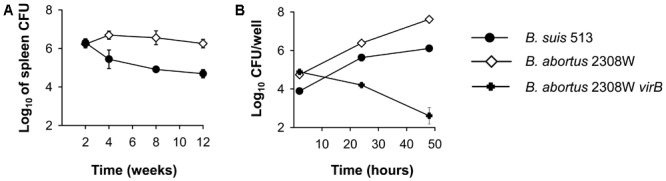
Multiplication of *B. abortus* 2308W and *B. suis* 513 in virulence models. **(A)** Bacterial multiplication in BALB/c mice. Each point is the mean ± standard deviation (*n* = 5) of the log CFU per spleen. **(B)** Intracellular multiplication in RAW 264.7 macrophages (the *B. abortus* 2308W *virB* mutant is used as a control of attenuation). Values are mean ± standard errors for triplicate infections and the results shown are representative of three independent experiments.

Since analysis of the *B. suis* 513 mutants in mice requires a definition of the parameters of virulence of the parental strain in this laboratory model and these have not been studied previously, we first inoculated BALB/c mice with 10^4^ and 10^5^ CFU of *B. suis* 513. These doses neither caused death nor triggered any signs of septic shock in the next days, a result similar to those that are characteristic of the *Brucella* species infecting domestic ruminants (Grilló et al., 2012). Therefore, we inoculated BALB/c mice intraperitoneally with 5 × 10^4^ CFU of *B. suis* 513 or *B. abortus* 2308W and determined the CFU numbers in spleen in the following weeks (**Figure 6A**). At week 2 (acute phase of infection; Grilló et al., 2012), *B. suis* 513 reached CFU numbers similar to those obtained with *B. abortus*. Thereafter, although CFU numbers were approximately 1.5 logs lower than those of *B. abortus* 2308W, *B. suis* 513 showed persistence typical of the chronic phase of classical *Brucella* species (Grilló et al., 2012). Virulence of *B. suis* 513 was confirmed in RAW 264.7 macrophages (**Figure 6B**).

**FIGURE 7:**
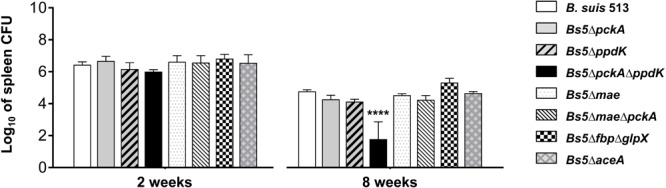
Bacterial loads in the spleens of BALB/c mice at 2 and 8 weeks post-infection of *B. suis* 513 and mutants *Bs5ΔpckA, Bs5ΔppdK, Bs5ΔpckAΔppdK, Bs5Δmae, Bs5ΔmaeΔpckA, Bs5ΔfbpΔglpX*, and *Bs5ΔaceA*. Statistical differences with *B. suis* 513 were significant at week 8 for *Bs5ΔpckAΔ*ppdK (^∗∗∗∗^*p* ≤ 0.0001).

Once we knew these characteristics of *B. suis* 513, we infected BALB/c mice with the above-described *B. suis* 513 mutants and determined the spleen CFU numbers in the acute and chronic phase of infection (i.e., 2 and 8 weeks after infection, respectively). We found that dysfunction of the genes of the gluconeogenic phosphatases Fbp and GlpX did not result in a decrease in the CFU numbers in the spleen of mice at either infection phase (**Figure 7**). Likewise, neither the *pckA* nor the *ppdK* mutant displayed CFU numbers different from the wild type control in either infection phase (**Figure 7**). On the other hand, the double *pckA*-*ppdK* mutant was markedly attenuated in the chronic phase (*p* ≤ 0.0001) to the extent that we did not detect any bacteria in the spleens of two of the five mice in this group (limit of detection of this method = 3.3 CFU/mL of the homogenized spleen; Grilló et al., 2012). Altogether, these results strongly suggest that PckA and PpdK are active during the infectious process, either alternatively or simultaneously. In keeping with this interpretation, partial complementation of the double mutant with plasmid pRH001-*ppdK* (pAZI-19; Supplementary Table S1) restored the ability to persist in the spleens of BALB/c mice (Supplementary Figure S3).

We did not find attenuation for Bs5Δ*mae* and Bs5Δ*mae*Δ*pckA* (**Figure 7**), which indicates that conversion of malate into pyruvate or oxaloacetate into phosphoenolpyruvate (**Figure 1**) is not essential for multiplication of *B. suis* 513 in BALB/c mouse spleen cells. Also, we did not observe attenuation for *Bs5ΔaceA* (**Figure 7**), meaning that the glyoxylate shunt is not essential either for *B. suis* 513 multiplication in the spleen of BALB/c mice.

## Discussion

Despite repeated isolation from wild rodents in the Caucasus provinces of the former USSR (Meyer, 1976; Vershilova et al., 1983; Corbel, 1984), there is little accessible information on the *in vitro* nutritional requirements and virulence of *B. suis* biovar 5 in standard brucellosis laboratory models. Remarkably, Russian workers described these isolates as displaying “luxurious” growth (Lyamkin et al., 1981) and amino acid oxidative abilities broader than those of *B. suis* biovar 1 (Lyamkin et al., 1982; Vershilova et al., 1983), and on these bases they proposed a new “serobiotype” (*B. murium*; Banai and Corbel, 2010), which was not accepted later at a time when the taxonomic status of *B. suis* seemed clear to the *Brucella* Taxonomy Subcommittee (Meyer, 1976; Vershilova et al., 1983; Corbel, 1984). Consistent with these reports and the proposal, the reference strain *B. suis* 513 displayed growth characteristics departing from those of the classical *Brucella* species, represented in the present work by *B. abortus* 2308W, and were in this regard more similar to the fast-growing species *B. microti*. Noteworthy, the natural hosts of *B. suis* biovar 5 and *B. microti* are wild rodents, rather than domestic livestock (Vershilova et al., 1983; Corbel, 1984; Scholz et al., 2008) and both are closer to the early diverging brucellae than *B. melitensis, B. abortus*, or the other *B. suis* biovars (Whatmore, 2009; Soler-Lloréns et al., 2016; Al Dahouk et al., 2017). Nevertheless, whereas the *B. microti* strain investigated so far (CCM 4915) is lethal at doses higher than 10^4^ CFU/mouse and is rapidly eliminated from the spleen at non-lethal doses (Jiménez de Bagüés et al., 2010, 2011), 10^5^ CFU of *B. suis* 513 neither caused the death of mice nor triggered any signs of septic shock, and the CFU/spleen profile showed the acute and chronic phases characteristic of *B. abortus, B. melitensis*, and among *B. suis* at least that of biovar 1. Although in a different animal model, this observation is in line with those of the Russian workers who found that the *B. suis* biovar 5 isolates were similar to *B. suis* biovar 1 in pathogenicity and subsequent pathomorphological changes in guinea pigs (Lyamkin et al., 1983). It has been pointed out previously that fast growth and lethality correlate in *B. microti* CCM 4915 (Jiménez de Bagüés et al., 2010). It could be that these are two non-causally connected features or that fast growth results in a rapid increase in *Brucella* pathogen-associated molecular pattern (PAMP)-bearing molecules that could reach lethal levels not attained at the same infectious dose by the slow-growing strains. Although we cannot rule out that the fast growth of *B. suis* 513 does not contribute to any extent to lower the CFU/spleen counts by bolstering innate immunity recognition, it is clear that, despite its proximity to *B. microti* in growth rates, host, and phylogenomic position, *B. suis* 513 ranks with the classical smooth *Brucella* species in lethality and ability to persist in mice (Jiménez de Bagüés et al., 2011; Grilló et al., 2012). These observations, which should be confirmed in additional strains, suggest differences in key PAMP-bearing molecules and, coherent with this hypothesis is the lack of reactivity of *B. microti* LPS in Western blot with monoclonal antibodies recognizing the core-lipid A of *B. melitensis, B. abortus*, and *B. suis* biovars 1, 2, and 5 (Zygmunt et al., 2012; R. Conde-Álvarez, A. Zúñiga-Ripa, S. Köhler, M. Iriarte, and I. Moriyón, unpublished observations). Indeed, the core-lipid A of the classical species bears the PAMP modifications implicated in reduced innate immunity recognition and lower septic shock lethality (Lapaque et al., 2005).

**FIGURE 8:**
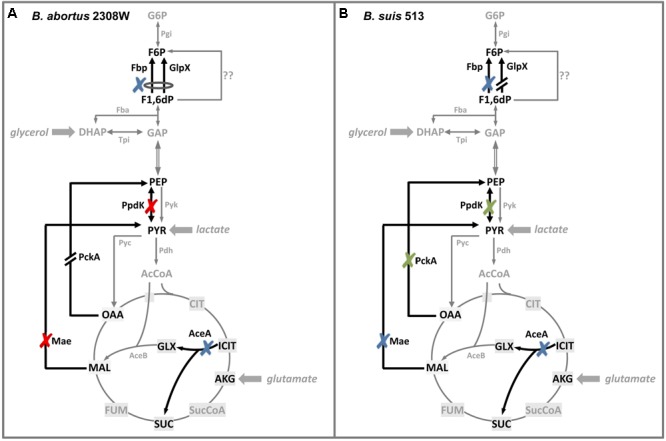
Summary of the results of the central C metabolism enzymes in *B. abortus* 2308W **(A)** and *B. suis* 513 **(B)**. Glycolysis, gluconeogenesis, TCA cycle, and glyoxylate shunt are shown. Black arrows indicate the steps studied in Zúñiga-Ripa et al. (2014) and in this work. Red crosses indicate attenuated mutants, green crosses specify attenuation when those deletions are combined, blue crosses show steps whose deletion does not affect virulence, broken arrows indicate inactive genes and question marks indicate undescribed enzymes/pathways (for abbreviations, see **Figure 1**).

*Brucella suis* 513 being closer to *B. abortus* 2308W in virulence in mice and macrophages, but more distant in growth rates in gluconeogenic media and in phylogenomic position, these two bacteria represent models suitable for comparing aspects of the central C metabolism of the core brucellae. The inability of *B. abortus* 2308W to grow on simple substrates as the only C source, most notably glucose, has to be interpreted with care, as this does not necessarily mean differences in central C pathways. The same inability has also been noted for the *B. melitensis* 16M strain kept in the laboratory of the authors, which requires methionine to grow on glucose and yet is fully virulent (Barbier et al., 2018). A similar explanation is likely to apply to *B. abortus* 2308W, as the differences with *B. suis* 513 *in vitro* disappear when glucose is combined with peptone (**Figure 2**). Indeed, these are aspects not related to the central C pathways in which there could be differences among strains with the same reference number kept in different laboratories, as it is the likely case of *B. abortus* 2308 variants (Suárez-Esquivel et al., 2016; see also below). The main differences and similarities that concern the C pathways specifically investigated here are summarized in **Figure 8**, and their potential significance is discussed below.

*In vitro, B. suis* 513 uses PpdK and PckA for phosphoenolpyruvate synthesis while *B. abortus* 2308W depends only on PpdK. This is likely to reflect the situation *in vivo*, because PpdK dysfunction causes attenuation of *B. abortus* 2308W in mice (Zúñiga-Ripa et al., 2014) but not in *B. suis* 513, in which attenuation occurred only in the double *pckA-ppdK* mutant. Although these observations strongly suggest that PckA is not essential for multiplication in the host and that, accordingly, it was not positively selected during evolution, we cannot presently extend this hypothesis to all core brucellae. On one hand, the genomes of all *B. abortus* and *B. melitensis* sequenced strains show the same *pckA* frameshift and subsequent stop codon that results in a truncated protein of 491 amino acids, and this is also true of the only *B. ovis* strain (BOV_2009 strain) sequenced. On the other, the *pckA* homologs of *B. suis* biovar 3 (strain 686), 4 (strain 40), or 5 (strain 513), *B. neotomae* (strain 5K33), *B. ceti* (strain B1/94), and *B. pinnipedialis* (strains M292/94/1 and M163/99/10) encode a protein of the same size (536 amino acids) as the *Agrobacterium tumefaciens* ortholog (for which there is evidence of PckA activity; Liu et al., 2005), *Ochrobactrum anthropi* and *Mesorhizobium loti*. Therefore, whereas the genomic, experimental, and epidemiological data coincide in indicating a role for PpdK but not for PckA in the virulence of *B. abortus* and *B. melitensis* in ruminants and accidental hosts like humans and canids, the interpretation of the conservation of *pckA* in other brucellae would require studies in the corresponding natural hosts.

The reasons for the conservation of PpdK over PckA in *B. abortus, B. melitensis*, and *B. ovis* are not obvious. It may be that, as opposed to the exclusively gluconeogenic role of PckA, the bidirectional nature of the PpdK catalyzed pyruvate ↔ phosphoenolpyruvate step makes the latter a more versatile enzyme (**Figure 8**). Although the direction of the C flow remains to be determined, it could indeed change during the life cycle of the bacteria depending on the substrates available at different stages, and in such a scenario *pckA* could become less useful and eventually superfluous. If TCA were the main source of precursors in the animal model, *B. suis* 513 could obtain phosphoenolpyruvate using Mae and PpdK and/or PckA (a possibility suggested by the *in vitro* growth with glutamate; **Figure 5**), and loss of PckA in *B. abortus* would not affect the Mae and PpdK route (able to supply phosphoenolpyruvate; **Figure 8**). This simple picture is in keeping with the attenuation of *B. abortus* 2308W *mae* mutants (Zúñiga-Ripa et al., 2014) but not with the virulence of the *B. suis* 513 double *mae*-*pckA* mutant (**Figure 7**) and the inability of *B. abortus* 2308W to grow with glutamate alone *in vitro* (**Table 1** and **Figure 3**). A hypothesis that could conciliate all the evidence available is that *B. abortus* is complementing the use of malate with a non-TCA substrate able to provide phosphoenolpyruvate, and that *B. suis* 513 uses such a substrate more efficiently, being in this way independent of malate and oxaloacetate. In this regard lactate is an attractive candidate because it can provide pyruvate (and phosphoenolpyruvate using PpdK; **Figure 8**). Moreover, whereas *B. abortus* 2308W (see below) cannot grow on lactate (**Figure 3**) and requires glutamate or glycerol as a complement *in vitro* (Zúñiga-Ripa et al., 2014), *B. suis* 513 can use lactate as the only source of C (**Figure 3**). Recently, on the basis of the availability of glutamate, lactate, and glycerol (and erythritol) in genital tissues, we have speculated that this set of simple C substrates could mimic the nutritional environment in the host (Letesson et al., 2017). It is thus conceivable that the dependence of *B. abortus* on at least two C sources and the loss of PckA reflect a progressive adaptation to such a nutritional environment. In this context, the recent work of Czyż et al. (2017) is also relevant, which described that *B. abortus* 2308 requires lactate dehydrogenase for intracellular survival in THP-1 monocytes. Interestingly, they also found that infected THP-1 monocytes increase glucose consumption and lactate production (a Warburg-like effect). However, the results reported by these authors on the ability of these bacteria to grow on substrates as the only C source do not clearly match those described here. Whereas they reported that a *B. abortus* 2308 strain was able to metabolize glucose, lactate, glutamate, and erythritol by measuring the reduction of a tetrazolium dye in a basal medium containing micromolar concentrations of arginine, glutamate, cystine, and 0.005% yeast extract, we did not find evidence for *B. abortus* 2308W use of lactate, glutamate, or glucose by a direct measurement of growth in mineral salts and vitamins. Although the discrepancies are difficult to interpret, and they could be due to the use of different basal media or strains (see above), it is important to note that the different ability of 2308W and the 2308 strain used by Czyż et al. (2017) to use lactate *in vitro* as the only C source do not contradict the hypothetical importance of lactate *in vivo*. The L-lactate permease and L-lactate dehydrogenase required for the metabolism of lactate (Barbier et al., 2018 and A. Zúñiga-Ripa, unpublished results) are conserved in all 2308 genomes sequenced and 2308W can use lactate when other substrates are added (Zúñiga-Ripa et al., 2014).

Whereas the experimental and genomic data on the role of PckA and PpdK in the core brucellae agree on the importance of the latter, this is not the case of AceA, the first enzyme of the glyoxylate shunt (**Figure 1**). Substrates such as fatty acids, some alcohols and esters, waxes, alkenes, and some methylated compounds enter central C metabolism at the level of acetyl-CoA, and this pathway enables some bacteria to use them as the sole C source (Caspi et al., 2018). The pathway may be active in some brucellae. Using a reporter system, we described that *B. abortus* 2308W expresses *aceA* at the beginning of the exponential phase in peptone-yeast extract-glucose but hardly in glutamate-lactate glycerol (Zúñiga-Ripa et al., 2014) and, in a proteomic study in J774 murine macrophages, Al Dahouk et al. (2008) found that *aceA* is expressed in *B. suis* 1330 (biovar 1) 48 h after infection. Also, Abdou et al. (2017) reported that a *B. suis* 1330 (biovar 1) mutant in the *regA* regulator overexpresses *aceA* after 3 days in glutamate-lactate-glycerol in an hypoxic persistence model. Using the *regA* mutant, its complemented strain and an *aceA* mutant grown in glutamate-lactate-glycerol supplemented with 0.05 mM sodium palmitate and 5 mM ammonium sulfate, these authors found evidence compatible with an active AceA by testing the respective cytosolic fractions with phenylhydrazine in the presence of isocitrate (Abdou et al., 2017). However, the evidence obtained in the analysis of *aceA* mutants in virulence models is not uniform. On one hand, infection of BALB/c mice with a *B. suis* 1330 *aceA* mutant results in lower CFU/spleen in the first 4 weeks but not at later times with respect to a complemented strain that did not fully restore virulence, and the differences are not reproduced in the liver (Abdou et al., 2017). On the other, we observed no attenuation for *B. suis* 513 or, in a previous work (Zúñiga-Ripa et al., 2014), for *B. abortus* 2308W in the spleens of BALB/c mice in the first 8 weeks. For *B. abortus* 2308W and *B. suis* 1330, the discrepancy has been attributed to strain differences (Abdou et al., 2017) and the results with *B. suis* 513 add to the hypothesis of a possible diversity of the brucellae at this level. There are small differences in the amino acid sequence of the respective AceA homologs (Supplementary Figure S4) but these are difficult to interpret without testing the activity of the purified proteins. It would be striking that AceA plays a role on virulence in some core brucellae but not in others, and further research is required to clarify these aspects of the central C metabolism of these bacteria. Thus far, the ability of *Brucella* to grow on fatty acids as the sole C source has not been explored (Gerhardt, 1958), possibly because of the early demonstration of their toxicity for *B. abortus* at very low (10–0.1 mg/L) concentrations (Boyd and Casman, 1951), which could hamper studies *in vitro*.

Both *B. abortus* 2308W and *B. suis* 513 double *fbp-glpX* mutants were able to grow in gluconeogenic media and, as discussed before (Zúñiga-Ripa et al., 2014), this suggests a hitherto undescribed gluconeogenic pathway (Caspi et al., 2018) possibly implicating a new type of phosphatase. Search for such an enzyme has been elusive (M. C. Durand-Steinhauser, T. Barbier, A. Zúñiga-Ripa, I. Moriyón, and J. J. Letesson, unpublished results) mainly because of the low activity of the hypothetical alternative gluconeogenic pathway in *B. abortus*, as the poor growth in gluconeogenic media reflects (**Figure 3** and **Table 1**). However, the double *fbp-glpX* mutant of *B. suis* 513 displays unscathed gluconeogenic ability in very simple media, a phenotype that is facilitating this investigation. Research in progress confirms the hypothesis that a new gluconeogenic enzyme compatible with a new pathway is in fact active in *B. suis* 513. Also worth commenting upon is the similar phenotype of wild type bacteria of *B. suis* 513 and *B. microti* CCM 4915 but not of *B. abortus* 2308W in gluconeogenic media (**Figure 1**). This simple observation suggests that gluconeogenesis is similar in the former two species, that the pathway is ancestral in the core brucellae and that there is a reduction of its efficiency in, at least, *B. abortus*. Such a reduction would be coherent with a reduced role or even non-essentiality of the pathway in the natural hosts of at least *B. abortus*, a hypothesis to be tested once the new pathway is fully elucidated.

## Author Contributions

AZ-R, JL, MI, and IM conceived the study. AZ-R and TB were the main researchers involved in the mutant and metabolic tests. LL-A, RC-Á, MdM, and PM contributed to mutant construction, growth measurements, and experiments in cells and mice. AZ-R and IM wrote the paper. All authors read and approved the manuscript content.

## Conflict of Interest Statement

The authors declare that the research was conducted in the absence of any commercial or financial relationships that could be construed as a potential conflict of interest.

## Footnotes

1 http://www.bacterio.net/-allnamesac.html
