# Supplementary material for: The Fast-Growing Brucella suis Biovar 5 Depends on Phosphoenolpyruvate Carboxykinase and Pyruvate Phosphate Dikinase but Not on Fbp and GlpX Fructose-1,6-Bisphosphatases or Isocitrate Lyase for Full Virulence in Laboratory Models
Source: Front Microbiol. 2018 Apr 5;9:641. doi: 10.3389/fmicb.2018.00641 (PMC5896264; doi:10.3389/fmicb.2018.00641)
Supplement: Supplementary file 1 [file Table_1.pdf]

TABLE S1. Bacterial strains and plasmids.

Strain or plasmid	Characteristics	Reference /Source
Brucella		
B. abortus 2308W	Nal^R spontaneous mutant of the 2308 strain received in 1983 from Dr. Lois M. Jones from the laboratory of Prof. David T. Berman (University of Wisconsin, Madison) as a lyophilized vial coming originally from the National Animal Disease Center at Ames, USA. It has been kept at the laboratory of the authors using the master seed strategy to minimize genetic drifts. It is virulent in mice and has been recently sequenced and found to differ from 2308 and 2308A (sequenced). Hence, the denomination "2308W" is adopted here.	(Sangari and Agüero, 1991; Suárez-Esquivel et al., 2016; Zúñiga-Ripa et al., 2014)
B. suis 513	Wild type, virulent, biotype 5, smooth LPS	(Vershilova et al., 1983)
B. microti CCM4915	Wild type, virulent, smooth LPS	(Scholz et al., 2008)
Bs5Δfbp	*B. suis* 513 *fbp* $_{\Delta 15\text{-}326}$	This work
Bs5ΔglpX	*B. suis* 513 *glpX* $_{\Delta 21\text{-}205}$	This work
Bs5ΔfbpΔglpX	*B. suis* 513 *fbp* $_{\Delta 15\text{-}326}$ *glpX* $_{\Delta 21\text{-}205}$	This work
Bs5ΔpckA	*B. suis* 513 *pckA* $_{\Delta 14\text{-}454}$	This work
Bs5ΔppdK	*B. suis* 513 *ppdK* $_{\Delta 35\text{-}859}$	This work
Bs5ΔpckAΔppdK	*B. suis* 513 *pckA* $_{\Delta 14\text{-}454}$ *ppdK* $_{\Delta 35\text{-}859}$	This work
Bs5Δmae	*B. suis* 513 *mae* $_{\Delta 26\text{-}700}$	This work
Bs5ΔmaeΔpckA	*B. suis* 513 *mae* $_{\Delta 26\text{-}700}$ *pckA* $_{\Delta 14\text{-}454}$	This work
Bs5ΔBMI_I149	*B. suis* 513 *BMI_I149* $_{\Delta 11\text{-}671}$	This work
Bs5ΔmaeΔBMI_I149	*B. suis* 513 *mae* $_{\Delta 26\text{-}700}$ *BMI_I149* $_{\Delta 11\text{-}671}$	This work
Bs5ΔaceA	*B. suis* 513 *aceA* $_{\Delta 11\text{-}419}$	This work
virB	*B. abortus* 2308W mutant deleted of *virB10*	(Sieira et al., 2000)
Escherichia coli		
S17-λ*pir*	Mating strain with plasmid RP4 inserted into the chromosome (Tpr Smr *rec*A *thi hsdRM+*, lambda *pir* phage lysogen RP4::2-Tc::Mu::Km Tn7)	(Miller and Mekalanos, 1988; Simon et al., 1983)
TOP10F´	F^- *lacI*q Tn *10* (Tetr) *mcrA* Δ(*mrr-hsdRMS-mcrBC*) 80*lac*ZΔM15 Δ*lacX74 recA1alaD139* Δ (*ara-leu*)*7697 galU galK rpsL endA1 nupG*	Invitrogen
**Plasmid**		
pCR2.1	Cloning vector, Km^R	Invitrogen
pJQKm	Suicide vector, Km^R, Sac^s	(Scupham and Triplett, 1997)
pRH001	Derivative of pMR10, Km^R; Cm^R	(Hallez et al., 2007)
pAZI-2	396 bp containing the *fbp* deletion allele cloned into pJQKm	(Zúñiga-Ripa et al., 2014)
pAZI-4	1150 bp containing the *glpX* deletion allele cloned into pJQKm	(Zúñiga-Ripa et al., 2014)
pAZI-6	538 bp containing the *pckA* deletion allele cloned into pJQKm	(Zúñiga-Ripa et al., 2014)
pAZI-8	516 bp containing the *aceA* deletion allele cloned into pJQKm	(Zúñiga-Ripa et al., 2014)
pMZI-2	508 bp containing the *ppdK* deletion allele cloned into pJQKm	(Zúñiga-Ripa et al., 2014)
pMZI-4	717 bp containing the *mae* deletion allele cloned into pJQKm	(Zúñiga-Ripa et al., 2014)
pAZI-19	Complete *ppdK* gene cloned into pRH001	(Zúñiga-Ripa et al., 2014)
pAZI-24	622 bp containing the BMI_I149 deletion allele, generated by PCR and cloned into pCR2.1	This work
pAZI-25	*Bam*HI/*Xba*I fragment from pAZI-24 cloned into the corresponding sites of pJQKm	This work

REFERENCES

Hallez, R., Letesson, J.-J., Vandenhaute, J., and de Bolle, X. (2007). Gateway-based destination vectors for functional analyses of bacterial ORFeomes: Application to the min system in *Brucella abortus*. *Appl. Environ. Microbiol.* 73, 1375–1379. doi:10.1128/AEM.01873-06.

Miller, V. L., and Mekalanos, J. J. (1988). A novel suicide vector and its use in construction of insertion mutations: Osmoregulation of outer membrane proteins and virulence determinants in *Vibrio cholerae* requires *toxR*. *J. Bacteriol.* 170, 2575–2583.

Sangari, F., and Agüero, J. (1991). Mutagenesis of *Brucella abortus*: comparative efficiency of three transposon delivery systems. *Microb. Pathog.* 11, 443–6.

Scholz, H. C., Hubalek, Z., Sedlacek, I., Vergnaud, G., Tomaso, H., Al Dahouk, S., et al. (2008). *Brucella microti* sp. nov., isolated from the common vole Microtus arvalis. *Int. J. Syst. Evol. Microbiol.* 58, 375–382. doi:10.1099/ijs.0.65356-0.

Scupham, A. J., and Triplett, E. W. (1997). Isolation and characterization of the UDP-glucose 4'-epimerase-encoding gene, *galE*, from *Brucella abortus* 2308. *Gene* 202, 53–59.

Sieira, R., Comerci, D. J., Sanchez, D. O., and Ugalde, R. A. (2000). A homologue of an operon required for DNA transfer in *Agrobacterium* is required in *Brucella abortus* for virulence and intracellular multiplication. *J. Bacteriol.* 182, 4849–4855. doi:10.1128/JB.182.17.4849-4855.2000.

Simon, L. D., Randolph, B., Irwin, N., and Binkowski, G. (1983). Stabilization of proteins by a bacteriophage T4 gene cloned in *Escherichia coli*. *Proc. Natl. Acad. Sci. U. S. A.* 80, 2059–62.

Suárez-Esquivel, M., Ruiz-Villalobos, N., Castillo-Zeledón, A., Jiménez-Rojas, C., Roop II, R. M., Comerci, D. J., et al. (2016). *Brucella abortus* Strain 2308 Wisconsin Genome: Importance of the Definition of Reference Strains. *Front. Microbiol.* 7, 1–6. doi:10.3389/fmicb.2016.01557.

Vershilova, P. A., Lyamkin, G. I., Malikov, V. E., and Dranovskaia, E. A. (1983). *Brucella* strains from mouse like rodents isolated in the USRR. *Int.J Syst.Bacteriol.* 33, 399–400.

Zúñiga-Ripa, A., Barbier, T., Conde-Álvarez, R., Martínez-Gómez, E., Palacios-Chaves, L., Gil-Ramírez, Y., et al. (2014). *Brucella abortus* depends on pyruvate phosphate dikinase and malic enzyme but not on Fbp and GlpX fructose-1,6-bisphosphatases for full virulence in laboratory models. *J. Bacteriol.* 196, 3045–3057. doi:10.1128/JB.01663-14.
