# Supplementary material for: The Fast-Growing Brucella suis Biovar 5 Depends on Phosphoenolpyruvate Carboxykinase and Pyruvate Phosphate Dikinase but Not on Fbp and GlpX Fructose-1,6-Bisphosphatases or Isocitrate Lyase for Full Virulence in Laboratory Models
Source: Front Microbiol. 2018 Apr 5;9:641. doi: 10.3389/fmicb.2018.00641 (PMC5896264; doi:10.3389/fmicb.2018.00641)
Supplement: Supplementary file 2 [file Image_1.PDF]

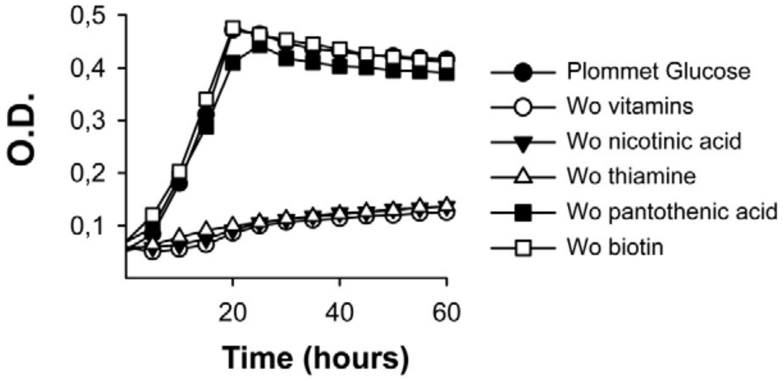

FIGURE S1. *B. suis* 513 growth in Plommet's medium with glucose and vitamins, this medium without (wo) vitamins (nicotinic acid, thiamine, pantothenic acid and biotin), nicotinic acid, thiamine, pantothenic acid or biotin. Each point represents the mean ± standard error of an experiment performed in technical triplicates (error bars are within the size of the symbols). The experiment was repeated at least three times with similar results.
