# Supplementary material for: The Fast-Growing Brucella suis Biovar 5 Depends on Phosphoenolpyruvate Carboxykinase and Pyruvate Phosphate Dikinase but Not on Fbp and GlpX Fructose-1,6-Bisphosphatases or Isocitrate Lyase for Full Virulence in Laboratory Models
Source: Front Microbiol. 2018 Apr 5;9:641. doi: 10.3389/fmicb.2018.00641 (PMC5896264; doi:10.3389/fmicb.2018.00641)
Supplement: Supplementary file 3 [file Image_2.PDF]

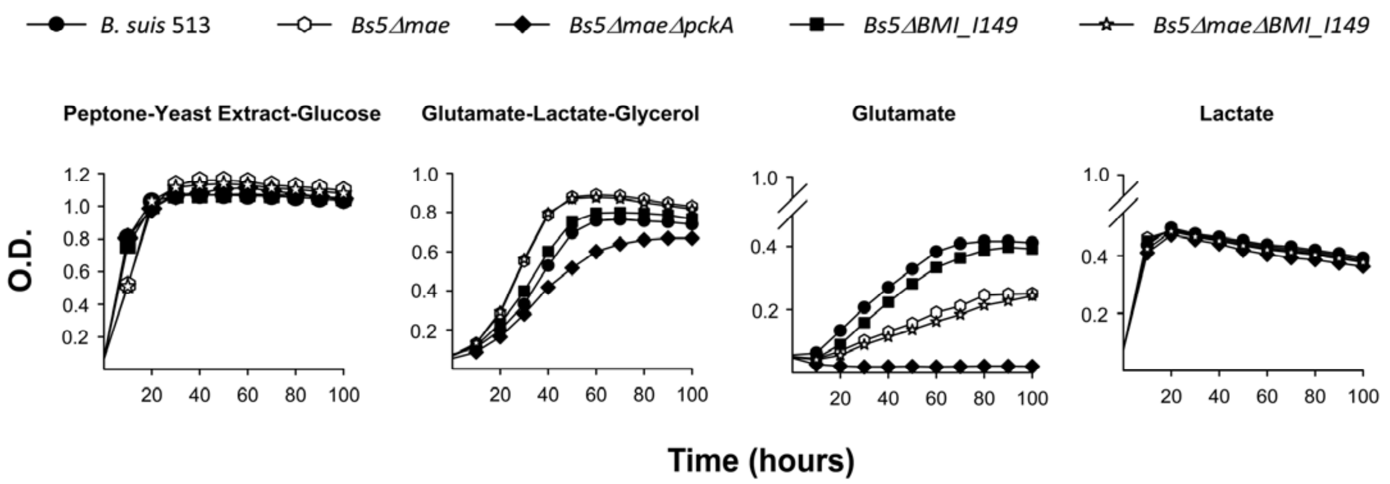

FIGURE S2. Growth in peptone-yeast extract-glucose, glutamate-lactate-glycerol, glutamate and lactate of *B. suis* strain 513 and mutants *Bs5∆mae, Bs5∆mae∆pckA, Bs5∆BMI_I149,* and *Bs5∆mae∆BMI_I149*. Each point represents the mean ± standard error (error bars are within the size of the symbols) of an experiment run in technical triplicates. The experiment was repeated three times with similar results.
