# Supplementary material for: The Fast-Growing Brucella suis Biovar 5 Depends on Phosphoenolpyruvate Carboxykinase and Pyruvate Phosphate Dikinase but Not on Fbp and GlpX Fructose-1,6-Bisphosphatases or Isocitrate Lyase for Full Virulence in Laboratory Models
Source: Front Microbiol. 2018 Apr 5;9:641. doi: 10.3389/fmicb.2018.00641 (PMC5896264; doi:10.3389/fmicb.2018.00641)
Supplement: Supplementary file 4 [file Image_3.PDF]

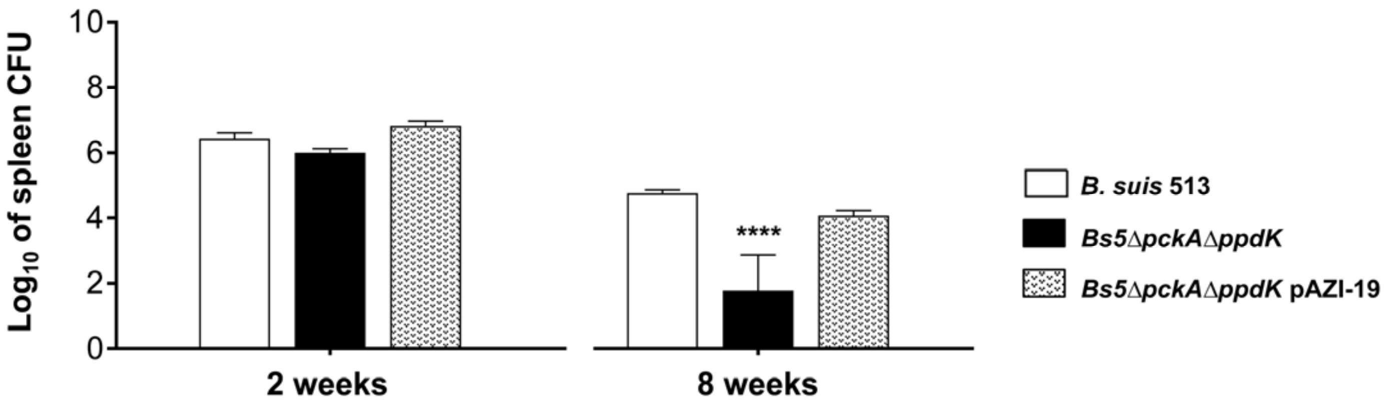

FIGURE S3. Bacterial loads in the spleens of BALB/c mice at 2 and 8 weeks post-infection of *B. suis* 513, mutant *Bs5ΔpckAΔppdK* and the complemented strain *Bs5ΔpckAΔppdK* pAZI-19 (pRH001-*ppdK*).
