# Supplementary material for: The Fast-Growing Brucella suis Biovar 5 Depends on Phosphoenolpyruvate Carboxykinase and Pyruvate Phosphate Dikinase but Not on Fbp and GlpX Fructose-1,6-Bisphosphatases or Isocitrate Lyase for Full Virulence in Laboratory Models
Source: Front Microbiol. 2018 Apr 5;9:641. doi: 10.3389/fmicb.2018.00641 (PMC5896264; doi:10.3389/fmicb.2018.00641)
Supplement: Supplementary file 5 [file Image_4.PDF]

A

```
B. suis 1330        MTDFYSLIPSAPKGRFDGIERAHTAEDVKRLRGSVEIKYSLAEMGANRLWKLIHEEDFVNALGALSGNQAMQMVRAGLKAIYLSGWQVAA
B. suis 513         MTDFYSLIPSAPKGRFDGIERAHTAEDVKRLRGSVEIKYSLAEMGANRLWKLIHEEDFVNALGALSGNQAMQMVRAGLKAIYLSGWQVAA
B. abortus 2308     MTDFYSLIPSAPKGRFDGIERAHTAEDVKRLRGSVEIKYSLAEMGANRLWKLIHEEDFVNALGALSGNQAMQMVRAGLKAIYLSGWQVAA
                    ******************************************************************************************

B. suis 1330        DANTASAMYPDQSLYPANAGPELAKRINRTLQRADQIETAEGKGLSVDTWFAPIVADAEAGFGGPLNAFEIMKAYIEAGAAGVHFEDQLA
B. suis 513         DANTASAMYPDQSLYPANAGPELAKRINRTLQRADQIETAEGKGLSVDTWFAPIVADAEAGFGGPLNAFEIMKAYIEAGAAGVHFEDQLA
B. abortus 2308     DANTASAMYPDQSLYPANAGPELAKRINRTLQRADQIETAEGKGLSVDTWFAPIVADAEAGFGGPLDAFEIMKAYIEAGAAGVHFEDQLA
                    ******************************************************************:***********************

B. suis 1330        SEKKCGHLGGKVLIPTAAHIRNLNAARLAADVMGTPTLIVARTDAEAAKLLTSDIDERDQPFVDYEAGRTAEGFYQVKNGIEPCIARAIA
B. suis 513         SEKKCGHLGGKVLIPTAAHIRNLNAARLAADVMGTPTLIVARTDAEAAKLLTSDIDERDQPFVDYEAGRTAEGFYQVKNGIEPCIARAIA
B. abortus 2308     SEKKCGHLGGKVLIPTAAHIRNLNAARLAADVMGTPTLIVARTDAEAAKLLTSDIDERDQPFVDYEAGRTAEGFYQVKNGIEPCIARAIA
                    ******************************************************************************************

B. suis 1330        YAPYCDLIWMETSKPDLAQARRFAEAVHKAHPGKLLAYNCSPSFNWKKNLDDATIAKFQCELGAMGYKFQFITLAGFHQLNYGMFELARG
B. suis 513         YAPYCDLIWMETSKPDLAQARRFAEAVHKAHPGKLLAYNCSPSFNWKKNLDDATIAKFQRELGAMGYKFQFITLAGFHQLNYGMFELARG
B. abortus 2308     YAPYCDLIWMETSKPDLAQARRFAEAVHKAHPGKLLAYNCSPSFNWKKNLDDATIAKFQRELGAMGYKFQFITLAGFHQLNYGMFELARG
                    *********************************************************** ******************************

B. suis 1330        YKDRQMAAYSELQQAEFAAEADGYTATKHQREVGTGYFDAMSLAITGGQSSTTAMKESTETAQFKPAAE
B. suis 513         YKDRQMAAYSELQQAEFAAEADGYTATKHQREVGTGYFDAVSLAITGGQSSTTAMKESTETAQFKPAAE
B. abortus 2308     YKDRQMAAYSELQQAEFAAEADGYTATKHQREVGTGYFDAVSLAITGGQSSTTAMKESTETAQFKPAAE
                    ****************************************:****************************
```

B

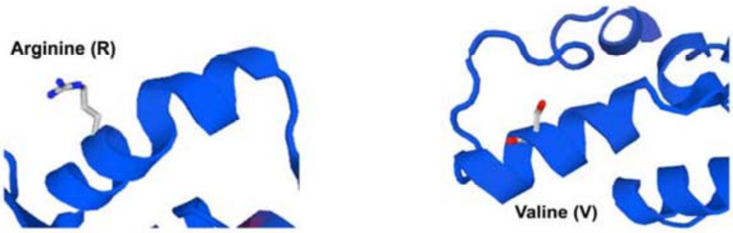

FIGURE S4. (A) Amino acid sequence alignment of AceA in *B. suis* 1330, *B. suis* 513 and *B. abortus* 2308. In bold and underlined, the 2 amino acids that differ in *B. suis* 1330 versus *B. suis* 513-*B. abortus* 2308. In position 330, a cysteine in *B. suis* 1330 is substituted by an arginine in the other 2 strains. In position 401, there is a methionine or valine in *B. suis* 1330 and *B. suis* 513-*B. abortus* 2308, respectively. (B) A Sequence-Based predicted structure built using SWISS-MODEL (Biasini et al., 2014) shows that both changes affect α-helices (*B. abortus* 2308 amino acids are shown). In the case of the substitution cysteine-arginine, the charge of the arginine might not be compensated destabilizing the structure. Moreover, the cysteine could be involved in disulfide bonds increasing the stability of the enzyme. Concerning the change valine-methionine the methionine present in *B. suis* 1330 may give higher stability to the α-helix.

REFERENCE

Biasini, M., Bienert, S., Waterhouse, A., Arnold, K., Studer, G., Schmidt, T., et al. (2014). SWISS-MODEL: modelling protein tertiary and quaternary structure using evolutionary information. *Nucleic Acids Res.* 42, W252–W258. doi:10.1093/nar/gku340.
